# Loss of GATA4 C-Terminus by p.S335X Mutation Modulates Coronary Artery Vascular Smooth Muscle Cell Phenotype

**DOI:** 10.1155/2021/3698386

**Published:** 2021-09-11

**Authors:** Ting-Yan Yu, Xin-Xin Chen, Qing-Wen Liu, Fang-Fang Ma, Hong-Lang Huang, Lei Zhou, Wei Zhang

**Affiliations:** ^1^Department of Cardiology, The First Affiliated Hospital of Nanjing Medical University, Nanjing, 210029 Jiangsu, China; ^2^Department of Echocardiography, The Second Affiliated Hospital of Zhejiang University, Hangzhou, 310009 Zhejiang, China; ^3^Department of Cardiology, The Affiliated Hospital of Jiangsu University, Zhenjiang, 212001 Jiangsu, China; ^4^Xiamen Key Laboratory of Cardiac Electrophysiology, Xiamen Institute of Cardiovascular Diseases, The First Affiliated Hospital of Xiamen University, School of Medicine, Xiamen University, Xiamen, 361003 Fujian, China

## Abstract

Coronary artery disease (CAD) has been the leading cause of morbidity and mortality worldwide, and its pathogenesis is closely related with the proliferation and migration of vascular smooth muscle cell (VSMC). We previously reported a truncated GATA4 protein lacking C-terminus induced by p.S335X mutation in cardiomyocyte from ventricular septal defect (VSD) patients. However, it is still unclear whether GATA4 p.S335X mutation could influence the development of CAD. GATA4 wild-type (WT) and p.S335X mutant (MU) overexpression plasmids were constructed and transfected transiently into rat coronary artery smooth muscle cell (RCSMC) to observe the proliferative and migratory abilities by MTS and wound healing assay, respectively. PCR array was used to preliminarily detect the expression of phenotypic modulation-related genes, and QRT-PCR was then carried out to verify the screened differentially expressed genes (DEGs). The results showed that, when stimulated by fetal bovine serum (10%) for 24 h or tumor necrosis factor-*α* (10 or 30 ng/ml) for 10 or 24 h, deletion of GATA4 C-terminus by p.S335X mutation in GATA4 enhanced the proliferation of RCSMC, without alteration of the migration capability. Twelve DEGs, including Fas, Hbegf, Itga5, Aimp1, Cxcl1, Il15, Il2rg, Il7, Tnfsf10, Il1r1, Irak1, and Tlr3, were screened and identified as phenotypic modulation-related genes. Our data might be beneficial for further exploration regarding the mechanisms of GATA4 p.S335X mutation on the phenotypic modulation of coronary VSMC.

## 1. Introduction

Coronary artery disease (CAD) is an atherosclerotic disease affecting the global human health and has been found to be the leading cause of death in both developed and developing countries [1]. Vascular smooth muscle cell (VSMC) is a major cell type presenting at all stages of atherosclerosis [2]. Unlike skeletal muscle cells or cardiomyocytes, VSMCs are not terminally differentiated and hence maintain phenotypic plasticity [3]. Under normal physiological conditions, VSMCs present as the contractile phenotype located in media and exhibit extremely low proliferative rate, as well as synthetic activity [4]. However, in the presence of environmental stimuli, such as inflammatory mediators, growth factors, and mitogens, VSMCs dedifferentiate into synthetic phenotype and lose the ability to contract, but migrate, proliferate, and accumulate into the intima [5]. VSMC dedifferentiation produces extracellular matrix and participates in fibrous cap formation [6] and subsequently accelerates the process of atherosclerosis ultimately [7].

The zinc finger transcription factor GATA4 belongs to an evolutionarily conserved GATA family, which consists of six members [8]. The importance of GATA4 is well appreciated in congenital heart diseases (CHDs) and some other cardiac malformations, such as myocardial hypoplasia, double outlets of the right ventricle, and common atrioventricular canal [9]. However, recent studies have found that GATA4 is also associated with the development of CAD. It has been revealed that GATA4 gene transcription was significantly enhanced in the peripheral blood mononuclear cells (PBMCs) in patients with severe stable CAD [10]. Further study suggested that, the higher expression of GATA4 was probably related to the increased GATA4 gene promoter activity affected by the DNA variants within its promoter in patients with acute myocardial infraction (AMI) [11]. Moreover, two single-nucleotide polymorphisms (SNPs), rs1062219 and rs804280, were unequivocally identified as risk variants for CAD, both of which were also linked to the development of CHDs [12]. We previously detected a p.S335X mutation in GATA4 in ventricular septal defect (VSD) patients, which could lead to truncated GATA4 protein lacking a conservative region at C-terminus [13]. The deletion of GATA4 C-terminus might induce VSD by suppressed cardiomyocyte proliferation and enhanced cell apoptosis [14]. Accordingly, we wonder whether the C-terminal deletion induced by p.S335X mutation in GATA4 also contribute to the development of CAD.

GATA4 was found to be an important regulator of coronary vasculature in the murine heart. It has been reported that conditional overexpression of GATA4 increased myocardial capillary and small conducting vessel densities, as well as increased coronary flow reserve [15]. Furthermore, the peri-infarct intramyocardial delivery of GATA4 vector prior to the ligation of left anterior descending coronary artery significantly increased the number of capillaries and reduced the infarct size [16]. GATA4 could not only target directly to the angiogenic factor vascular endothelial growth factor-A (VEGF-A) to promote angiogenesis but also interact with the transcriptional regulator friend of GATA2 (FOG2). GATA4-FOG2 would synergistically regulate a broad panel of angiogenesis-related genes to promote the formation of coronary vascular plexus [17]. Besides, GATA4 has also been revealed to participate in the regulation of VSMC proliferation and migration [18, 19]. However, the possible role of GATA4 p.S335X in the proliferation and migration of VSMC in coronary artery has not been studied so far. In the present study, a rat coronary artery smooth muscle cell (RCSMC) culture model overexpression p.S335X mutant of GATA4 was established in vitro. The proliferation and migration of RCSMC was validated, and the altered expression of important phenotypic modulation-related genes was assessed.

## 2. Materials and Methods

### 2.1. Cell Culture and Transient Transfection

RCSMCs (BNCC, Beijing, China) were cultured in DMEM (Gibco, Grand Island, NY, USA) supplemented with 10% fetal bovine serum (FBS; US origin, Gibco, Grand Island, NY, USA) and 1% penicillin and streptomycin (MP Biomedicals, Solon, OH, USA) at 37°C in a humidified atmosphere of 5% CO_2_. RCSMC in each well was transfected using Lipofectamine 2000 reagent (Invitrogen, Carlsbad, CA, USA) according to manufacturer's protocol.

### 2.2. Western Blot

Western blot samples were lysed within RIPA buffer (Pierce, Rockford, IL, USA) containing protease inhibitors. Then, the same amount of proteins was resolved by SDS-PAGE and PVDF membranes (Millipore, Bedford, MA, USA). After blocking with 5% skim milk for one hour, the blots were incubated with primary antibody GATA4 (Abcam, Cambridge, UK) overnight and then with horseradish peroxidase-conjugated secondary antibody for another one hour. Finally, protein bands were visualized using the Western blotting detection kit (Millipore, Bedford, MA, USA) and quantified with Image Pro Plus version 6 software (Media Cybernetics, Rockville, MD, USA).

### 2.3. QRT-PCR

Total RNA was extracted from RCSMC by Buffer RZ (Tiangen, Beijing, China) and then reversed into cDNAs using Transcriptor Fast Quant RT Kit (Tiangen, Beijing, China) following manufacturer's protocol. The obtained cDNAs were then mixed with primers and Fast SYBR Green Master Mix (Applied Biosystems, Vilnus, Lithuania) to carry out QRT-PCR by ABI 7500 PCR machine (Applied Biosystems, Foster City, CA, USA). Data was analyzed using 2^−∆∆Ct^ method and normalized to Rat Actb expression. The primers were listed in supplemental Table S1 and all primers were synthesized by Sangon Biotech (Shanghai, China).

### 2.4. MTS Assay

Cell proliferation assay was conducted by using CellTiter 96® Aqueous One Solution Cell Proliferation kit (MTS, Promega, USA). After 24-hour transfection and 4-hour starvation, RCSMCs were seeded at (2 − 3) × 103 cells/well in 96-well plates and then incubated at 37°C in a humidified atmosphere of 5% CO_2_ overnight. After cultivation in 100 *μ*l medium containing different drug concentrations for 10 or 24 h, 20 *μ*l MTS were added into each well and incubated in cell incubator for 4 hours. The absorbance was read at 490 nm on a microplate reader (Infinite M1000 Pro, Tecan, Switzerland).

### 2.5. Wound Healing Assay

After transfection, RCSMCs were seeded in 6-well plates at 2 × 10^7^ cells/well and incubated at 37°C in a humidified atmosphere of 5% CO_2_ overnight. When the degree of cell fusion reached above 90%, wounds were produced by a sterile 200 *μ*l plastic pipette tips. Cells were further cultured with medium containing different drug concentrations and allowed to migrate into the denuded area for 24 h. Images were acquired by microscope (Leica, Germany) at 4 × 40 magnification.

### 2.6. PCR Array Analysis

The total RNA samples were extracted by RNeasy Plus Mini Kit (Qiagen, Helden, Germany) according to manufacturer's protocol. cDNA synthesis was performed on 1 *μ*g RNA in a 10 *μ*l sample volume using the RT^2^ First Strand Kit (Qiagen, Frederick, MD, USA) as recommended by manufacturer's protocol. Then, the obtained cDNAs were mixed with RT^2^ SYBR Green qPCR Mastermix (Qiagen, Frederick, MD, USA) to perform RT^2^ Profiler PCR Array (Qiagen, Frederick, MD, USA) by ABI-7500 machine (Applied Biosystems, Foster City, CA, USA). All data from the PCR array experiments were analyzed by Qiagen GeneGlobe Data Analysis Center Web Portal (https://geneglobe.qiagen.com/cn/analyze).

### 2.7. Statistical Analysis

Data were expressed as means ± SEM from at least three independent experiments. Differences between groups were analyzed by Prism 6 software (GraphPad, La Jolla, CA, USA). One-way ANOVA with Fisher's LSD test was used for comparison of data in more than two groups. *P* < 0.05 was considered to be statistically significant.

## 3. Results

### 3.1. Establishment of RCSMC Culture Model Overexpressing GATA4 WT and MU

**Figure 1:**
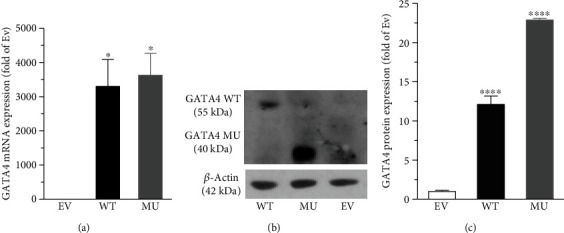
The transfection of plasmids pcDNA6-GATA4-WT and pcDNA-GATA4-MU. (a) QRT-PCR results demonstrated the successful transfection and expression of GATA4 mRNA in RCSMCs. One-way ANOVA, ^∗^*P* < 0.05 versus EV, *n* = 3. (b) Western blot results showed the expression of GATA4 in WT group (55 KDa) and MU group (40 KDa, truncated form). (c) Semiquantitative analysis of Western blot results. One-way ANONA, ^∗∗∗∗^*P* < 0.0001 versus EV, *n* = 3.

RCSMC is a kind of long spindle cell, with adherent growth and typical characteristics of smooth muscle cell. Plasmids pcDNA6-GATA4-WT and pcDNA-GATA4-MU have been constructed, representing GATA4 full length sequence and GATA4 p.S335X mutant sequence. The latter could result in deletion of GATA4 C-terminus [13]. The plasmids were transfected into RCSMC for 24 h to establish GATA4 WT and MU overexpression models. QRT-PCR and Western blot were then used to evaluate the transfection efficiency. QRT-PCR results showed that the expression of GATA4 mRNA in WT and MU groups was significantly higher than that in GATA4 empty vector (EV) group (*P* < 0.05, Figure 1(a)), indicating the successful transfection of GATA4 WT and MU plasmids. Western blot results demonstrated that deletion of GATA4 C-terminus induced by p.S335X mutation decreased the molecular weight of target protein in MU group (40 KDa) compared to that in WT (55 KDa), i.e., the GATA4 protein was truncated (Figures 1(b) and 1(c)).

### 3.2. Deletion of GATA4 C-Terminus Enhanced RCSMC Proliferation

**Figure 2:**
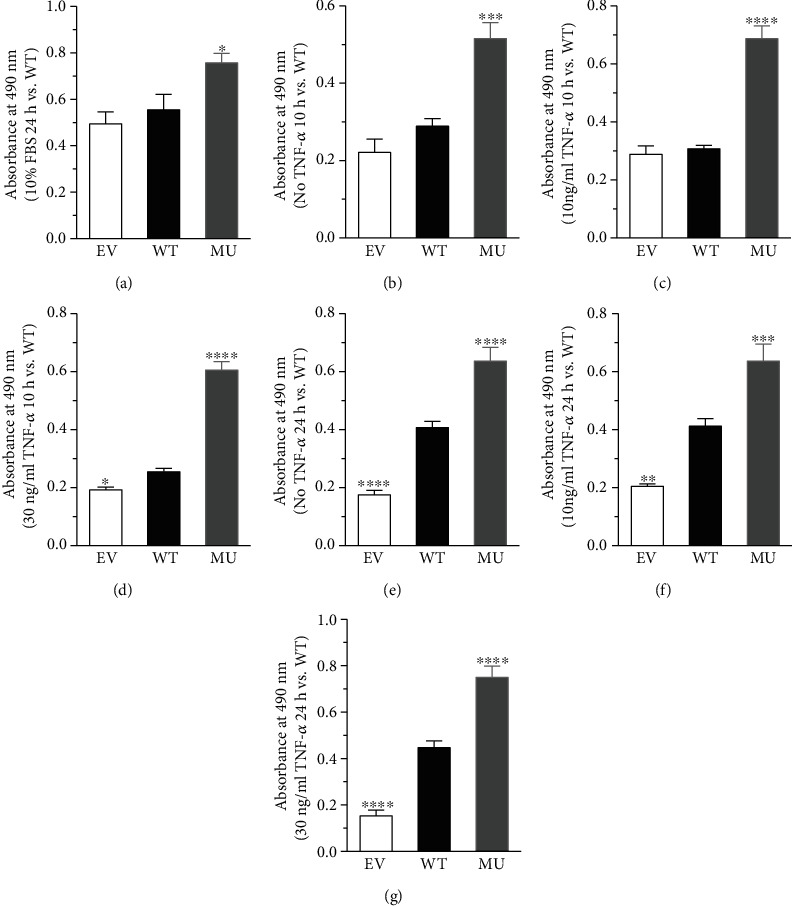
Deletion of GATA4 C-terminus enhanced RCSMC proliferation. The effects of GATA4 WT and MU overexpression on RCSMC proliferation was detected by MTS. The cells were treated with (a) 10% FBS for 24 h, (b–d) TNF-*α* (10 or 30 ng/ml) for 10 h, and (e–g) TNF-*α* (10 or 30 ng/ml) for 24 h. One-way ANOVA, ^∗^*P* < 0.05, ^∗∗∗^*P* < 0.001, ^∗∗∗∗^*P* < 0.0001 versus WT, *n* = 3.

MTS assay kit was used to detect the viability of RCSMC. The results showed that the viability of RCSMC in MU group in the presence of 10% FBS was significantly higher than that in WT group (*P* < 0.05, Figure 2(a)), suggesting enhanced proliferation of cells in MU group.

To further confirm the effect of GATA4 C-terminal deletion on the proliferation capability of RCSMC, we adopted different concentrations of the proinflammatory cytokine tumor necrosis factor-*α* (TNF-*α*, 10 and 30 ng/ml) under serum-free condition. Cells in WT group showed higher viability compared to that in EV group in case of TNF-*α* 10 ng/ml for 24 h, and 30 ng/ml for 10 and 24 h. Impressively, C-terminal deletion by p.S335X mutation (MU) in GATA4 further enhanced the viability of cells compared to that in WT group regardless of the TNF-*α* concentration (10 or 30 ng/ml) or duration of drug administration (10 or 24 h) (*P* < 0.05, Figures 2(b)–2(g)). These data suggested that the C-terminus of GATA4 might exert a negative regulatory effect on RCSMC proliferation, whose deletion may play a vital role in RCSMC proliferation.

### 3.3. Migration of RCSCM Was Not Significantly Modulated by GATA4 MU

**Figure 3:**
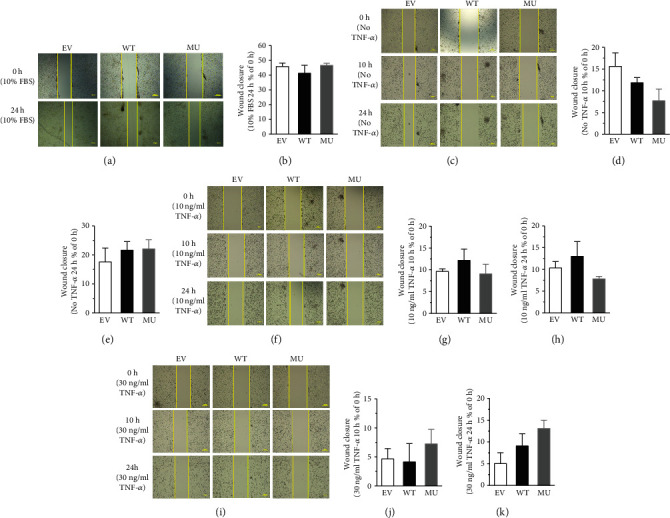
Deletion of GATA4 C-terminus did not alter RCSMC migration. Wound healing assay was adopted to observe the migratory rate of RCSMC. The quantitative analysis of wound healing assay was presented by the wound closure rate relative to the initial distance. Magnification: 4 × 40. (a, b) Representative images of migrated cells incubated with 10% FBS taken at 0 and 24 h after injury. One-way ANOVA, *P* > 0.05 versus WT, *n* = 3. (c–k) Representative images of migrated cells stimulated by TNF-*α* (10 or 30 ng/ml) taken at 0, 10, and 24 h after injury. One-way ANOVA, *P* > 0.05 versus WT, *n* = 4.

Wound healing assay was performed to evaluate the migration ability of RCSMC in GATA4 WT, MU and, EV groups. The results showed that, after additional 10% of FBS to the culture medium for 24 h, the wound healing rates of cells in WT, MU, and EV groups were all around 45%, with no significant difference between the groups, respectively (Figures 3(a) and 3(b)). Neither did the administration of TNF-*α* (10 and 30 ng/ml) for 10 or 24 h induce significant changes in the migration of RCSMCs in the present settings (Figures 3(c)–3(k)).

### 3.4. Screening and Verification of Phenotypic Modulation-Related Genes Regulated by the Deletion of GATA4 C-Terminus

**Figure 4:**
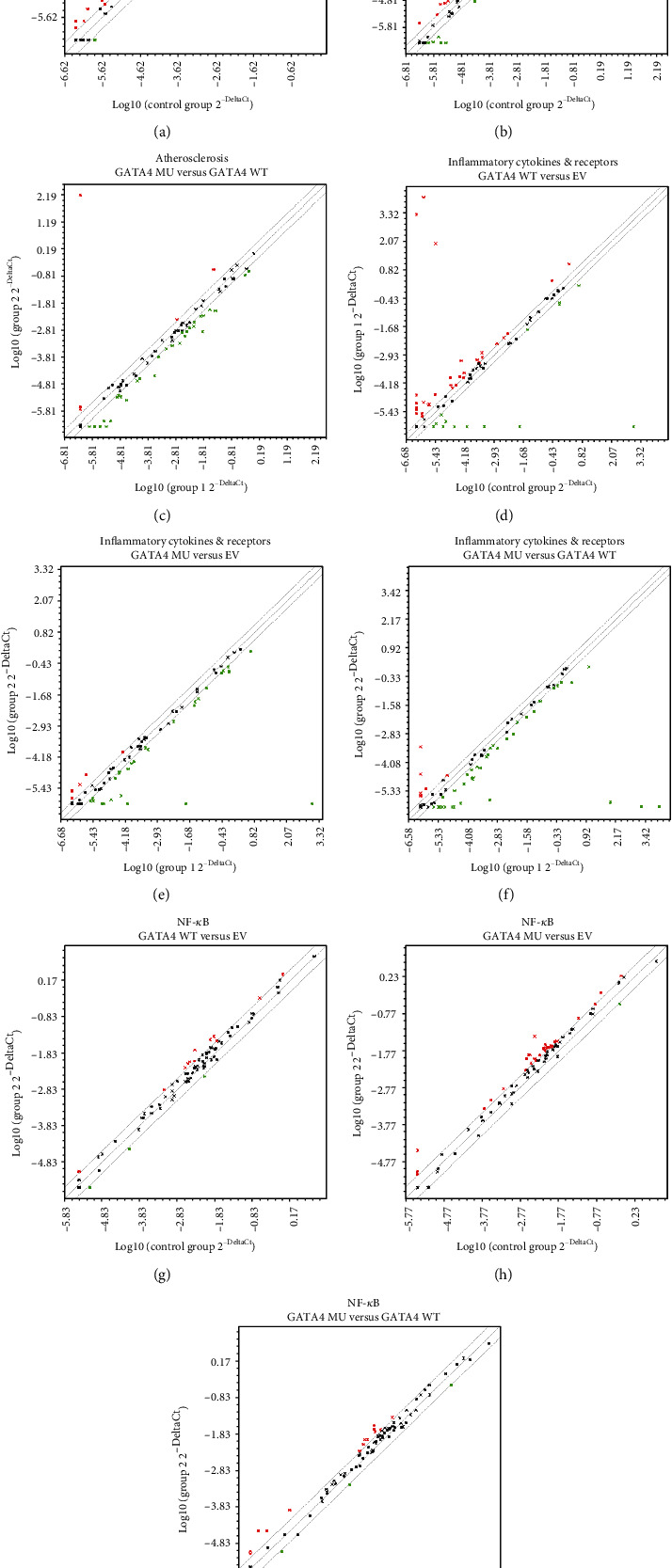
The scatter plots of PCR array results. (a–c) Rat atherosclerosis; (d–f) rat inflammatory cytokines and receptors; (g–i) rat NF-*κ*B. Fold change ≥ 2 was taken as the threshold value. (a, d, and g) WT versus EV; (b, e, and h) MU versus EV; (c, f, and i) MU versus WT.

Since p.S335X mutation-induced GATA4 C-terminal deletion showed potent effects on the proliferation of RCSMC, a high-throughput PCR analysis (RT^2^ Profiler RT-PCR Array) was performed to detect the expression profile of phenotypic modulation-related genes. The scatter plots taking 2 folds of gene regulation as the threshold value are shown in Figure 4 ((a–c), rat atherosclerosis PCR array (PARN-038Z); (d–f), rat inflammatory cytokines and receptors PCR array (PARN-011Z); and (g–i), rat nuclear factor-*κ*B (NF-*κ*B) pathway PCR array (PARN-025Z), respectively).

**Figure 5:**
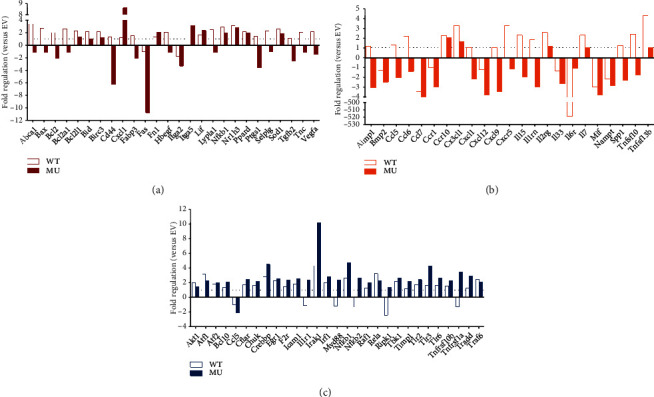
The fold regulation of differential expression genes (DEGs) in WT and MU groups compared to EV group. (a) Rat atherosclerosis-related DEGs. (b) Rat inflammatory cytokines and receptor-related DEGs. (c) Rat NF-*κ*B pathway-related DEGs.

Subsequently, differentially expressed genes (DEGs) in WT and MU groups in comparison with EV group were screened and listed in Figure 5. In rat atherosclerosis PCR array (Figure 5(a)), Abca1, Bax, Bcl2, Bcl2a1, Cd44, Fabp3, Hbegf, Lypla1, Ptgs1, Selplg, Tgfb2, Tnc, and Vegfa had reverse trend between WT and MU groups, while the trends of Bcl2l1, Bid, Birc3, Cxcl1, Fas, Fn1, Itga2, Itga5, Lif, Nfkb1, Nr1h3, Ppard, and Sod1 were consistent. In rat inflammatory cytokines and receptors PCR array (Figure 5(b)), the DEGs with opposite tendency were Aimp1, Ccl5, Ccl6, Cxcl1, Cxcl9, Cxcr5, Il15, Il1rn, Spp1, and Tnfsf10, while the DEGs on the same trend were Bmp2, Ccl7, Ccr1, Ccr10, Cx3cl1, Cxcl12, Il2rg, Il33, Il6r, Il7, Mif, Nampt, and Tnfsf13b. In rat NF-*κ*B pathway PCR array (Figure 5(c)), Akt1, Atf1, Atf2, Bcl10, Cflar, Chuk, Crebbp, Egr1, F2r, Icam1, Irak1, Irf1, Nfkb1, Raf1, Rela, Tbk1, Timp1, Tlr2, Tlr3, Tlr6, Tnfrs10b, Tradd, and Traf6 were with the consistent tendency, while Ccl5, Il1r1, Myd88, Nfkb2, Ripk1, and Tnfrsf1a had opposite trend.

**Figure 6:**
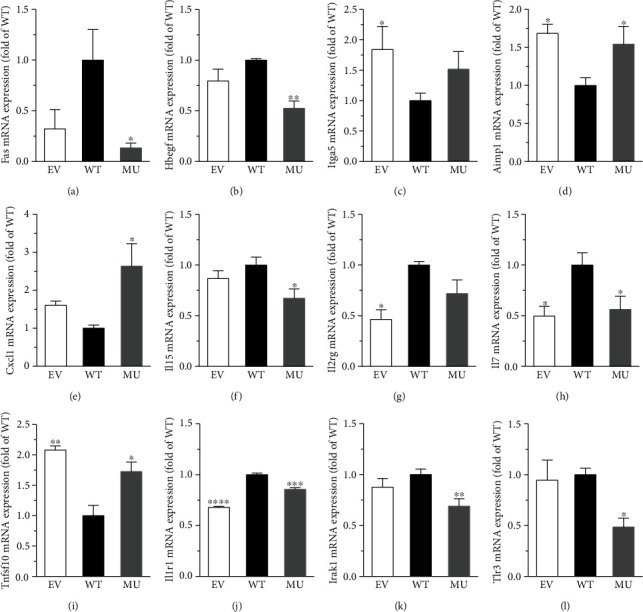
QRT-PCR verification of the screened DEGs. (a) Fas; (b) Hbegf; (c) Itga5; (d) Aimp1; (e) Cxcl1; (f) Il15; (g) Il2rg; (h) Il7; (i) Tnfsf10; (j) Il1r1; (k) Irak1; (l) Tlr3. One-way ANOVA, ^∗^*P* < 0.05, ^∗∗^*P* < 0.01, ^∗∗∗^*P* < 0.001, ^∗∗∗∗^*P* < 0.0001 versus WT, *n* = 7.

As per the previous reports, we further selected part of the DEGs which are closely linked to the phenotypic modulation of VSMC and verified their expression by using QRT-PCR (Figure 6). Particularly, the expression of Fas and Hbegf was markedly lower in MU group compared to that in WT group. Aimp1, Cxcl1, and Tnfsf10 had higher expression in MU group compared to that in WT group, while the expression of Il15 and Il7 was lower. Analogously, the expression of Il1r1, Irak1, and Tlr3, was decreased in MU group versus WT group. These data suggested that the above-mentioned genes might be regulated by GATA4 C-terminus.

## 4. Discussion

In the present study, the deletion of GATA4 C-terminus by p.S335X mutation enhanced RCSMC proliferation, without alteration of migration in the cell culture model in vitro. Twelve DEGs including Fas, Hbegf, Itga5, Aimp1, Cxcl1, Il15, Il2rg, Il7, Tnfsf10, Il1r1, Irak1, and Tlr3 were screened and identified as phenotypic modulation-related genes that might be regulated by GATA4 and its C-terminus. Our data could provide a clue for further exploration for the molecular mechanisms of GATA4 on the modulation of VSMC phenotype.

Previously, GATA4 has been proven to regulate the proliferation capability of a variety of cell types, such as cardiomyocytes, small intestinal epithelial cells, follicular granulosa cells, and leukemia lymphocytes [20–24]. However, regarding the VSMC, only two studies concerning human pulmonary artery SMCs and mouse aortic SMCs were reported so far [18, 19]. Since the proliferation of VSMC is a vital phenotypic modulation characteristic in VSMC dedifferentiation and is closely linked to atherosclerosis [5, 7], we wondered if GATA4 account for the development of CAD via regulating the proliferation of coronary artery VSMC. Hence, we firstly adopted RCSMC overexpressing GATA4 WT or MU as cell culture model. The expression level of MU protein was higher than WT (Figure 1(c)), possibly because of the different plasmids used or the difference in transfection efficacy. The cell viability of RCSMC was significantly increased by MU in the presence of different stimuli (10% FBS for 24 h, or TNF-*α* 10 or 30 ng/ml for 10 or 24 h) (Figure 2), demonstrating that the deletion of GATA4 C-terminus by p.S335X mutation could enhance proliferation of RCMSC. It is likely that GATA4 protein could exert bidirectional regulatory effects on cell proliferation, i.e., the C-terminus of GATA4 could possibly protect VSMC against TNF-*α*-induced proliferation to some extent. Such findings extended the current knowledge about the function of GATA4 C-terminus, whose deletion not only promoted the apoptosis of cardiomyocyte [13, 14], and also enhanced the proliferation of RCSMC that might contribute to the development of CAD.

Migration is another important characteristic of phenotypic modulation in the process of VSMC dedifferentiation. Our data showed that the migratory rates of RCSMCs among WT, MU, and EV groups yielded no significant difference in the presence of different stimuli (10% FBS for 24 h, or TNF-*α* 10 or 30 ng/ml for 10 or 24 h) (Figure 3), indicating the ineffectiveness of GATA4 C-terminal deletion on RCSMC migration under such experimental conditions. Rabinovitch and co-authors reported that the migration of pulmonary artery SMC could be stimulated by S100A4/Mts1 depending on the phosphorylation of GATA4 [18]. Previous studies found that 10% FBS and TNF-*α* 10-100 ng/ml were efficient for the migration of aortic VSMC [25–28]. However, in our experiments, neither WT nor MU exerted significant effect on the migratory capability of RCSMC and that might be resulted from the different cell types.

To further explore the possible molecules involved in the effects of GATA4 C-terminal deletion on RCSMC proliferation, a panel of phenotypic modulation-related genes was screened by using PCR array. Subsequently, QRT-PCR was performed to confirm the changed expression of the following: Fas, Hbegf, Itga5, Aimp1, Cxcl1, Il15, Il2rg, Il7, Tnfsf10, Il1r1, Irak1, and Tlr3. These genes are considered to be associated with atherosclerosis and vascular inflammatory response during the pathogenesis of cardiovascular disorders including CAD.

Fas, also known as CD95 or APO-1, is a member of the TNF/nerve growth factor superfamily and can induce cell proliferation by stimulating the formation of death-inducing signaling complex (DISC). The latter activates the mitogen-activated protein kinase (MAPK) family, as well as NF-*κ*B. In addition, Fas ligands can also induce ligand-dependent epidermal growth factor (EGF) receptor activation and phosphorylation, triggering cell proliferation through extracellular-signal regulated kinases (ERK)1/2 signaling pathway [29]. Hbegf belongs to the EGF family and is known to induce VSMC proliferation by autophosphorylation of EGF receptors, followed by the activation of PI3K-Akt, MAPK, and janus kinase-signal transducer and activator of transcription (JAK-STAT) pathways [30, 31]. Itga5, a member of integrin *α* chain family, binds predominantly with integrin *β*1 and functions in cell surface adhesion and signaling [32]. It has been reported that Itga5 could enhance the proliferation of human periodontal ligament stem cells (PDLSCs) via PI3K-Akt and MEK1/2/ERK1/2 pathways [33]. As per our QRT-PCR results (Figure 6), the expressions of Fas and Hbegf were both lower in MU group compared to that in WT group, implying that the deletion of GATA4 C-terminus might inhibit their expressions. Additionally, Itga5 expression was higher in EV group and was not significantly different in WT and MU group, indicating that the C-terminus of GATA4 was not required for the regulation of Itga5.

Aimp1 is a cytokine involved in the regulation of angiogenesis, immune activation, and fibroblast proliferation. Aimp1 could promote the proliferation of human bone marrow-derived mesenchymal stem cells by activating the *β*-catenin/TCF complex via FGFR2-mediated activation of Akt [34]. Another study focused on intimal hyperplasia and found a significant upregulation of Aimp1, as well as Cxcl1, at the early stage of arterial injury in rat model [35]. Cxcl1, also known as growth-related oncogene protein-*α* (GRO-*α*), is a chemotactic cytokine. TNF-*α* could stimulate Cxcl1 release from human umbilical vein endothelial cells (HUVECs) through JNK-mediated Cxcl1 mRNA expression and p38 MAPK- and PI3K-mediated Cxcl1 secretory processes, and the recombinant Cxcl1 secreted by HUVECs enhanced cell proliferation in turn [36]. Conversely, another study pointed out that recombinant Cxcl1 induced by cyclic mechanical stretch (CMS) in a JNK-dependent manner failed to impact the proliferation of rat aortic smooth muscle cells (RASMCs) [37]. Tnfsf10, also named after TRAIL, is a member of a subset of the TNF receptor superfamily [38]. It has been reported that Tnfsf10 could promote the proliferation of VSMC via the activation of ERK1/2, Akt, and NF-*κ*B [39–41]. Il15, Il7, and Il2rg are different subtypes of interleukins. Il15 has been detected in atherosclerosis plaques as a proinflammatory cytokine and could attenuate SMC proliferation possibly via inhibiting the chemokine receptor CX3CR1 [42, 43]. Il7 is a hematopoietic factor secreted by mesenchymal cells in the bone marrow and thymus, whose variant Il7*δ*5 could induce human breast cancer cell proliferation and cell cycle progression in a PI3K-Akt-dependent manner [44]. IL2RG encoded by Il2rg is an important signaling component of many cytokine receptors, including those of Il1, Il4, Il7, Il9, Il15, and Il21. A case report in Japan showed that, an atypical *γ*c deficiency in IL2RG might be related with the occurrence of EBV-associated *γδ* T-cell lymphoproliferative disorder [45]. In our study, the expression of Aimp1, Cxcl1, and Thfsf10 was significantly higher in MU groups than that in WT groups, while the trends of Il15 and Il7 were opposite, suggesting that GATA4 C-terminus might target to the above-mentioned genes. Moreover, Il2rg expression was lower in EV group and was comparable between WT ang MU groups, implying that GATA4 might not regulate the expression of Il2rg through its C-terminus.

Encoded by Il1r1, IL-1 receptor 1 (IL-1R1) protein could be bound by Il-1*β* to recruit the molecular adaptor myeloid differentiation primary response protein 88 (MyD88) and thus induce IL-1, IL-6, and TNF-*α* synthesis through NF-*κ*B activation. It has been reported that the IL-1R1/MyD88 signaling was involved in pulmonary vessel remodeling [46]. Gomez et al. observed smaller lesions nearly devoid of SMC and a fibrous cap in SMC-specific Il1r1 KO mice [47], indicating the close relationship between Il1r1 and atherosclerosis. Protein IRAK1 encoded by Irak1 has been reported to regulate VSMC proliferation in carotid arteries with IRAK4 via the TLR4/NF-*κ*B signaling pathway [48]. Protein kinase C (PKC)-*ε*-IRAK–ERK axis also played important roles in the process of VSMC proliferation and neointimal hyperplasia mediated by IRAK1 [49]. Tlr3 is a member of the TLR family and can bind to double-strand RNA to regulate vascular remodeling [50]. It could evoke a proinflammatory and proliferative phenotype in human VSMC, probably mediated by ERK1/2 and NF-*κ*B signaling [51]. The expression of Il1r1, Irak1, and Tlr3 in MU group was lower than that in WT group, indicating that the deletion of GATA4 C-terminus could suppress their expressions.

**Figure 7:**
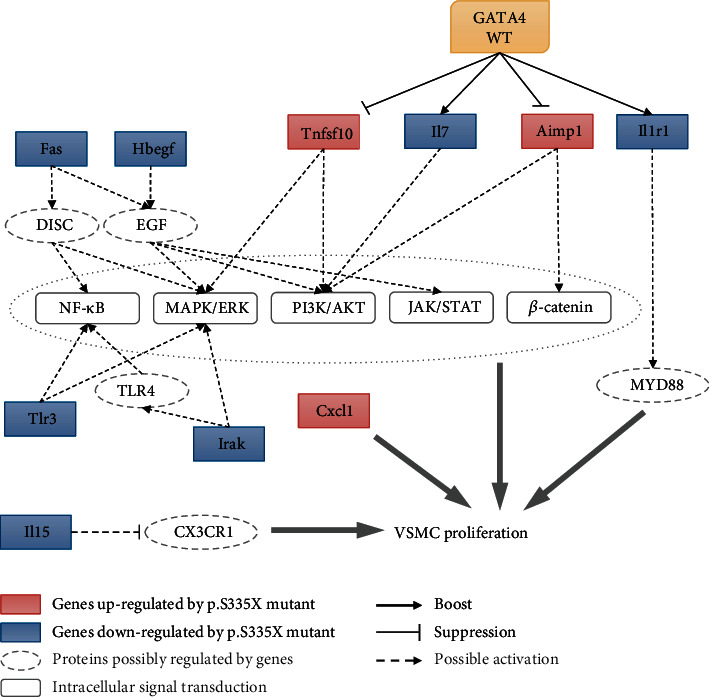
A schema illustrating the potential relationship between the regulation of RCSMC phenotype by GATA4 WT and MU and the DEGs and possible intracellular signals.

The above-mentioned DEGs regulated by the deletion of GATA4 C-terminus had the potential to regulate the proliferation of VSMC or other cell types through MAPK family (P38, JNK1/2, and ERK1/2), NF-*κ*B, PI3K-Akt, and JAK-STAT pathways. A schema was provided in Figure 7 in order to illustrate the potential relationship between the regulation of RCSMC phenotype by GATA4 WT and MU and the DEGs and possible intracellular signals. However, whether such pathways are responsible for the phenotypic modulation of RCSMC by GATA-4 C-terminus still remained unclear. Further in-depth mechanism studies are required to elucidate the exact signaling pathway(s) that account for the effects of GATA4 under both in vitro and in vivo conditions.

## 5. Conclusions

We herein reported that the deletion of GATA4 C-terminus induced by p.S335X mutation could enhance the proliferation but not the migration of RCSMC in vitro under certain stimulation. Twelve DEGs, including Fas, Hbegf, Itga5, Aimp1, Cxcl1, Il15, Il2rg, Il7, Tnfsf10, Il1r1, Irak1, and Tlr3, were screened and identified as phenotypic modulation-related genes that might be targeted by GATA4 and its C-terminus in the regulation of RCSMC proliferation. Our findings could provide a new sight and might be beneficial for the further mechanism study concerning CAD associated with GATA4 mutation.

## Data Availability

The data used to support the findings of this study are available from the corresponding author upon request.

## Supplementary Materials

Supplementary Materials Supplemental Table S1: primer sequences for QRT-PCR.
