# Supplementary material for: Loss of GATA4 C-Terminus by p.S335X Mutation Modulates Coronary Artery Vascular Smooth Muscle Cell Phenotype
Source: Mediators Inflamm. 2021 Sep 11;2021:3698386. doi: 10.1155/2021/3698386 (PMC8449727; doi:10.1155/2021/3698386)
Supplement: Supplementary Materials — Supplemental Table S1: primer sequences for QRT-PCR. [file 3698386.f1.docx]

Loss of GATA4 C-terminus by p.S335X mutation modulates coronary artery vascular smooth muscle cell phenotype

Ting-Yan Yu, Xin-Xin Chen, Qing-Wen Liu, Fang-Fang Ma, Hong-Lang Huang, Lei Zhou, Wei Zhang

Supplemental Material

Table S1. Primers sequences for QRT-PCR.

| Gene | Primer | Sequence (5'- to -3') |
| --- | --- | --- |
| Gata4 | Forward | GACACCCCAATCTCGATATGTTTG |
|  | Reverse | ACAGATAGTGACCCGTCCCA |
| Bax | Forward | ACAGATCATGAAGACAGGGGC |
|  | Reverse | AGCCATCCTCTCTGCTCGAT |
| Bcl2a1 | Forward | TATCCACTCCCTGGCTGAGA |
|  | Reverse | CTTGGAGCCGATTCAAAGGC |
| Bid | Forward | CCAAGTGTCGGTCGGCAAAC |
|  | Reverse | TGCTGACCTCAGAGTCCATGA |
| Cxcl1 | Forward | ACTCAAGAATGGTCGCGAGG |
|  | Reverse | ACGCCATCGGTGCAATCTAT |
| Fabp3 | Forward | GTCGGTACCTGGAAGCTAGT |
|  | Reverse | CAAAGCCCACACCGAGTGA |
| Fas | Forward | ACGGGCACCAAAATGAAAGC |
|  | Reverse | CTGGCAAAAAGAACACGCCA |
| Hbegf | Forward | GGCTACTTCTGAGATGGCGG |
|  | Reverse | CCCAGTCAGGGTAGCAACTG |
| Itga2 | Forward | ACAAGGCAACTGGCTACTGG |
|  | Reverse | CGTCTCCCATTCGGTTGTCA |
| Itga5 | Forward | CATGAAGGCAGGCACCAGTA |
|  | Reverse | TGGGAAGGAGACCATGTTGC |
| Tgfb2 | Forward | GTGGGCAGCTTTTGCTCCATA |
|  | Reverse | GCGGACGATTCTGAAGTAGGG |
| Tnc | Forward | GAATTGCTCCCAGCATCCGTA |
|  | Reverse | TTCCGGTTCAGCTTCTGTGG |
| Aimp1 | Forward | GTGTGACTCGGTCTGCATCA |
|  | Reverse | CGTGATACGTCGACAGGCTT |
| Bmp2 | Forward | GAATGGACGTGCCCCCTAGT |
|  | Reverse | CACCATGGTCGACCTTTAGGA |
| Cxcl12 | Forward | GGACTTTCCAGTAGACCCCTG |
|  | Reverse | CAAATCTCAGCATGACCCCAG |
| Cxcl9 | Forward | TCTGCCATGAAGTCCGTTGC |
|  | Reverse | CCTCGAACTCCACAGTGATCC |
| Il15 | Forward | TGAACTGCTTTCTCCTGGAAT |
|  | Reverse | GTGCTGTTTGCAAGGTAGAGC |
| Il2rg | Forward | TGGTGTGTCTAAAGGGCTGAC |
|  | Reverse | CTCACTGACGTGGCAGAACC |
| Il7 | Forward | TTTCAGACGGCACACAAACAC |
|  | Reverse | TTTCTTGGAGGTTGCTACTACAT |
| Spp1 | Forward | AGCCAGCCAAGGACCAACTA |
|  | Reverse | GCTGTAATGCGCCTTCTCCT |
| Tnfsf10 | Forward | GACAGTCTCGAAGGACGGAG |
|  | Reverse | GCAGTATGGGATCGGGGTAG |
| Crebbp | Forward | TCATTGCTGGTGACTCATGC |
|  | Reverse | TCAGCTCGTTATCGGGACAC |
| Il1r1 | Forward | CCTCTGCCTCTTGACGATGG |
|  | Reverse | AGGACGTGCGGCAAGTATAG |
| Irak1 | Forward | ATCAAGCCAAGCCCAGAGAG |
|  | Reverse | ACCACCCTCTCCAATCCTGA |
| Myd88 | Forward | CCCCGAAGGCTTTCTACCAA |
|  | Reverse | GTTTTTGTGTGTCCGCTGCT |
| Nfkb1 | Forward | GAACAATGCCTTCCGGCTGAG |
|  | Reverse | GCCTCTGTGTAGCCCATCTGT |
| Timp1 | Forward | CTGCAACTCGGACCTGGTTA |
|  | Reverse | CAGCGTCGAATCCTTTGAGC |
| Tlr3 | Forward | CTACAACAGCCTCCGCGAC |
|  | Reverse | ACAGATACTTCAGGTGCGGG |
| Tradd | Forward | TCAGCTCATAGTGAACCGGC |
|  | Reverse | GATCCCTCAGTGCTCGACAG |
